# The Effect of Tai Chi on Cardiorespiratory Fitness for Coronary Disease Rehabilitation: A Systematic Review and Meta-Analysis

**DOI:** 10.3389/fphys.2017.01091

**Published:** 2018-01-04

**Authors:** Ying-li Yang, Ya-hong Wang, Shuo-ren Wang, Pu-song Shi, Can Wang

**Affiliations:** ^1^First Clinical Medical School, Beijing University of Chinese Medicine, Beijing, China; ^2^Department of Cardiology, Dongzhimen Hospital, Beijing University of Chinese Medicine, Beijing, China

**Keywords:** Tai Chi, coronary disease, rehabilitation, cardiorespiratory fitness, VO_2_max, systematic review, meta-analysis, CRD42017063773.

## Abstract

**Background:** Tai Chi that originated in China as a martial art is an aerobic exercise with low-to-moderate intensity and may play a role in cardiac rehabilitation.

**Aim:** To systematically review the effect of Tai Chi on cardiorespiratory fitness for coronary disease rehabilitation.

**Methods:** We performed a search for Chinese and English studies in the following databases: PubMed, EMBASE, Cochrane Central Register of Controlled Trials, Chinese Biomedical Literature Database, China Knowledge Resource Integrated Database, Wanfang Data, and China Science and Technology Journal Database. The search strategy included terms relating to or describing Tai Chi and coronary disease, and there were no exclusion criteria for other types of diseases or disorders. Further, bibliographies of the related published systematic reviews were also reviewed. The searches, data extraction, and risk of bias (ROB) assessments were conducted by two independent investigators. Differences were resolved by consensus. RevMan 5.3.0 was used to analyze the study results. We used quantitative synthesis if the included studies were sufficiently homogeneous and performed subgroup analyses for studies with different control groups. To minimize bias in our findings, we used GRADEpro to grade the available evidence.

**Results:** Five studies were enrolled—two randomized controlled trials (RCTs) and three nonrandomized controlled trials (N-RCTs)—that included 291 patients. All patients had coronary disease. ROB assessments showed a relatively high selection and detection bias. Meta-analyses showed that compared to other types of low- or moderate-intensity exercise, Tai Chi could significantly improve VO_2_max [MD = 4.71, 95% CI (3.58, 5.84), *P* < 0.00001], but it seemed less effective at improving VO_2_max as compared to high-intensity exercise. This difference, however, was not statistically significant [MD = −1.10, 95% CI (−2.46, 0.26), *P* = 0.11]. The GRADEpro showed a low level of the available evidence.

**Conclusion:** Compared to no exercise or other types of exercise with low-to-moderate intensity, Tai Chi seems a good choice for coronary disease rehabilitation in improving cardiorespiratory fitness. However, owing to the poor methodology quality, more clinical trials with large sample size, strict randomization, and clear description about detection and reporting processes are needed to further verify the evidence.

## Introduction

According to WHO statistics, coronary disease is the leading cause of death globally, more than even the sum of all cancer-related deaths (World Health Organization, 2017). In recent years, with advances in cardiac rehabilitation, coronary disease mortality in developed countries has been significantly reduced (Mozaffarian et al., 2016).

Cardiac rehabilitation has a 55-year-old development history, and meta-analyses show that exercise-based cardiac rehabilitation can reduce all-cause fatality rate by 8–37% and reduce cardiac fatality rates by 7–38% in patients with coronary disease (Anderson et al., 2016; Taylor et al., 2017). Cardiorespiratory fitness, or aerobic capacity, which is often reflected by VO_2_max, is one of the main concerning parameters in cardiac rehabilitation. Low aerobic capacity is a strong predictor of cardiac and all-cause mortality. Even a small increase in VO_2_max can improve the functional level in activities of daily living, therefore leading to better quality of life (Lan et al., 2013). Tai Chi, a Chinese martial art form, is a proven aerobic exercise with low-to-moderate intensity (Smith et al., 2015; Hui et al., 2016), which likely also has potential benefits in cardiac rehabilitation. Because Tai Chi is simple and easy to learn and practiced not only in China (Zheng, 2004; Liu, 2016; Sun and Lu, 2017), other developed countries have also gradually incorporated Tai Chi for cardiac rehabilitation research (Dunlop, 2011; Lee and Koo, 2011; Lan et al., 2013; Nery et al., 2015; Wieczorrek et al., 2016). Nery (Nery et al., 2014), Dalusung-Angosta (Dalusung-Angosta, 2011), and Ng (Ng et al., 2012) have conducted systematic reviews of randomized controlled trials (RCTs) of Tai Chi intervention on coronary disease. The results suggest that Tai Chi can be used as adjuvant therapy for coronary disease, thereby playing a role in cardiorespiratory fitness improvement. However, some of the shortcomings of these RCTs include a small sample size and relatively low methodological quality, notably lack of randomization and possible detection and reporting bias. Further, the above-mentioned systematic reviews were all conducted in the Western population 3–6 years ago, and the included literature were all in English publications. Therefore, it is necessary to update the systematic review and include Chinese studies conducted by Chinese researchers to comprehensively and systematically evaluate the effect of Tai Chi on cardiorespiratory fitness in patients with coronary disease and provide evidence-based data for the development of cardiac rehabilitation.

## Methods

We registered our protocol of this systematic review and meta-analysis on PROSPERO in advance (No. CRD42017063773, http://www.crd.york.ac.uk/PROSPERO).

### Inclusion criteria

1. Participants: There were no restrictions on the patients' age, gender, disease duration, case source, nationality, or race. In the original literature, patients should have had a clear diagnosis of coronary disease, while the rehabilitation period did not have any restriction. Moreover, there were no restrictions with respect to other types of diseases or disorders.
2. Intervention: The original literature should have provided clear descriptions about Tai Chi: the style (such as Yang's style, Chen's style, 24 styles, or 48 styles); the teaching and practicing process (e.g., taught by professional guider); and the practice frequency (e.g., 3–4 times a week, 30 min every week).
3. Control: Considering the fact that Tai Chi is a practice, the control could be a blank control, which means no exercise or positive control such as walking, jogging, or stretching; therefore, any kind of exercise was acceptable.
4. Outcomes: Primary outcomes were cardiorespiratory fitness assessed by VO_2_max, which was tested by the cardiopulmonary exercise test (CPET). Secondary outcomes were vital signs and adverse events.
5. Study type: Clinical studies with treatment for more than 1 month. Owing to the specificity of Tai Chi intervention and the fact that the preliminary search had found only few strictly conducted RCTs of Tai Chi for coronary disease, we expanded the scope of the included study types to RCTs, non-randomized controlled trials (N-RCTs), as well as cohort studies and case-control studies.

### Literature searches

We performed a search for Chinese and English studies in the following databases: PubMed, EMBASE, Cochrane Central Register of Controlled Trials (CENTRAL), Chinese Biomedical Literature Database (CBM), China Knowledge Resource Integrated Database (CNKI), Wanfang Data, and China Science and Technology Journal Database (VIP). The search strategy included terms relating to or describing Tai Chi and coronary disease. Studies published between the day since the database was established and April 2017 were retrieved. Bibliographies of the related published systematic reviews were also reviewed. An illustrative PubMed search strategy is shown below:

#1 ((((((((((Coronary Disease [MeSH Terms]) OR Coronary Diseases) OR Disease, Coronary) OR Diseases, Coronary) OR Coronary Heart Disease) OR Coronary Heart Diseases) OR Disease, Coronary Heart) OR Diseases, Coronary Heart) OR Heart Disease, Coronary) OR Heart Diseases, Coronary)
#2 ((((((((((((((Myocardial Infarction [MeSH Terms]) OR Infarction, Myocardial) OR Infarctions, Myocardial) OR Myocardial Infarctions) OR Cardiovascular Stroke) OR Cardiovascular Strokes) OR Stroke, Cardiovascular) OR Strokes, Cardiovascular) OR Heart Attack) OR Heart Attacks) OR Myocardial Infarct) OR Infarct, Myocardial) OR Infarcts, Myocardial) OR Myocardial Infarcts)
#3 ((((Angina Pectoris [MeSH Terms]) OR Stenocardia) OR Stenocardias) OR Angor Pectoris)
#4 ((((((((((Myocardial Ischemia [MeSH Terms]) OR Ischemia, Myocardial) OR Ischemias, Myocardial) OR Myocardial Ischemias) OR Ischemic Heart Disease) OR Heart Disease, Ischemic) OR Disease, Ischemic Heart) OR Diseases, Ischemic Heart) OR Heart Diseases, Ischemic) OR Ischemic Heart Diseases)
#5 #1 OR #2 OR #3 OR #4
#6 (((((((((((Tai Ji [MeSH Terms]) OR Tai Chi Chuan) OR T'ai Chi) OR Taijiquan) OR Taiji) OR Quan, Tai Ji) OR Ji Quan, Tai) OR Tai Ji Quan) OR Chi, Tai) OR Tai Chi) OR Tai-ji)
#7 #5 AND #6

First, two independent investigators reviewed the titles and abstracts. Abstracts that did not meet the eligibility criteria were excluded, and those that did not provide sufficient information about the inclusion criteria were further reviewed. Next, the same investigators analyzed the full texts, blinded to each other's review. Differences between the reviewers were resolved by consensus.

### Data extraction

Two investigators independently performed data extraction by using the pre-piloted standardized forms. The collected data included: basic information (study ID, database, document type, author, publishing year, study type); methodological characteristics risk of bias (ROB); participants' demographic details (diagnostic criteria, inclusion criteria, exclusion criteria, sample size, age, gender, nationality, and disease duration); interventions (group numbers, intervention descriptions, control descriptions); outcomes, fall outs; results of outcomes; and others (foundations, conflicts of interests, ethical review, and important citations). All differences were resolved by consensus.

### Risk of bias assessment

Two investigators independently assessed the methodological quality of the included studies by using RevMan 5.3.0, according to Cochrane Handbook criteria for judging ROB in the “Risk of bias” assessment tool. The judgment on the risk of bias was categorized as low, unclear, or high ROB in the following aspects: random sequence generation (selection bias), allocation concealment (selection bias), blinding of participants and personnel (performance bias), blinding of outcomes assessment (detection bias), incomplete outcome data (attrition bias), selective reporting (reporting bias), and other bias. Again, all differences were resolved by consensus.

### Statistical analyses

RevMan 5.3.0 provided by the Cochrane Collaboration was used to analyze the results of the studies. All outcomes were continuous variables, so we expressed them as mean ± standard deviation and then calculated the mean difference (MD) and obtained the two-sided *P*-value and 95% confidence interval (CI). We used the complete case data as the analysis data. Heterogeneity between the studies in effect measures was assessed using both the Chi-squared test and the I-squared statistic with an I-squared value >50% indicative of substantial heterogeneity. We used quantitative synthesis if the included studies were sufficiently homogeneous, both statistically and clinically. When the I-squared value was lower than 30% and *P*-value > 0.10, a fixed-effect model was used; otherwise, a random effects model was used.

As the meta-analysis of primary outcomes showed significant heterogeneity, we performed separate subgroup analyses for studies with different control groups. Since the methodological quality of the included studies was generally low, and the results of the subgroup analyses showed significant positive results, we did not carry out further sensitivity analyses.

Because we included only five studies, we did not generate a funnel plot to detect publication bias. To minimize bias in our findings and recommendations, we used the GRADEpro online summary of findings table for outcomes to grade the available evidence. The evaluation included bias risk, inconsistency (heterogeneity), indirect, imprecision, and publication bias; the level of each evidence was graded as very low, low, moderate, or high.

## Results

### Literature screening

**Figure 1:**
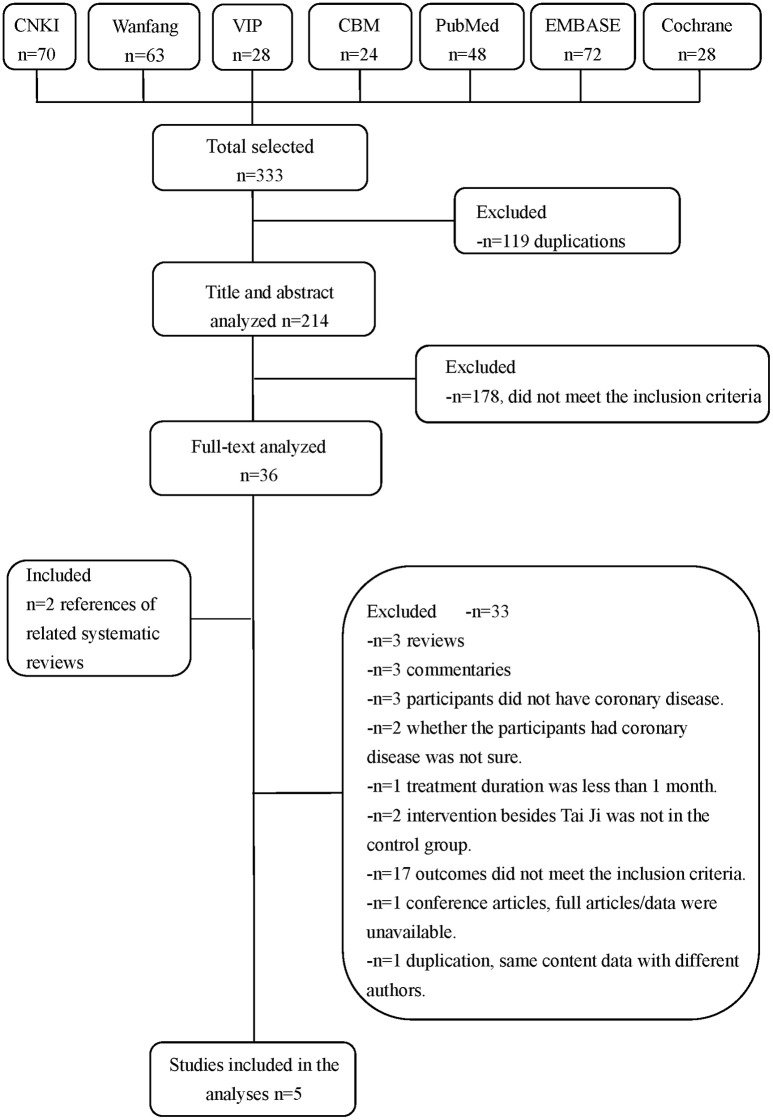
Literature screening process.

We retrieved 333 original literatures from electronic bibliographic databases published between 1983 and 2017. After 119 duplications were excluded, we screened the titles and abstracts of 214 publications, whereby another 178 papers that did not meet the inclusion criteria were excluded. We downloaded the full text of the remaining 36 publications for future screening, of which we included two literatures from the bibliographies of the related published systematic reviews. Finally, five articles met the inclusion criteria and were completely analyzed (Figure 1).

### Characteristics of included studies

The five studies were published between 1999 and 2015: two were RCTs and three were N-RCTs, and the studies' sample sizes were relatively small. Patients included in these studies were from China, Taiwan (China), and Brazil. Coronary disease included post coronary artery bypass grafting (CABG), post percutaneous coronary intervention (PCI), chronic stable angina, and the recovery period of acute myocardial infarction (AMI). Most studies considered Yang's style of Tai Chi as the intervention and had sessions by professional, experienced guiders who taught low-to-moderate intensity Tai Chi. Controls included blank, walking, jogging, and stretching. Outcomes included VO_2_max/VO_2_peak and peak heart rate (HR). The treatment duration lasted from 12 weeks to 1 year (Table 1).

**Table 1:** Characteristics of included studies.

**Studies**	**Coronary disease type**	**Nationality**	**Intervention group**			**Control group**			**Outcomes**	**Study type**	**Treatment duration**
			**Sample size**	**Age**	**Intervention**	**Sample size**	**Age**	**Control**			
Lan et al., 1999	Post-CABG	Taiwan (China)	9	55.7 ± 7.1	Yang's style, teaching guider, 54 min every morning	11	57.2 ± 7.6	Walking 3 times a week	VO_2_ peak HR peak	N-RCT	One year
Chang et al., 2010	Post-PCI/CABG	Taiwan (China)	22	58.2 ± 11.3	Yang's style, teaching guider, at least 3 times a week, 50 min each time	32	63.3 ± 9.4	Blank	HR peak	N-RCT	6 months
Wang et al., 2010	Chronic stable angina	China	42 27 22	67.9 ± 6.8 68.7 ± 7.0 65.5 ± 6.9	Simplified 24 style:low intensity−30 min/d, 3 d/w, moderate intensity−40 min/d, 5 d/w, Jogging:high intensity−40 min/d, 5 d/w	24	69.5 ± 4	Blank	VO_2_ max HR max	N-RCT	6 months
Nery et al., 2012	AMI	Brazil	18	59 ± 10	Yang's style, teaching guider, 3 times a week, 60 min each time	23	58 ± 9	Stretching 2 times a week	VO_2_ max	RCT	12 weeks
Nery et al., 2015	AMI	Brazil	31	56 ± 9	“Beijing” style, 3 times a week, 60 min each time	30	60 ± 9	Stretching 3 times a week	VO_2_ peak HR peak	RCT	12 weeks

### Risk of bias assessment

**Figure 2:**
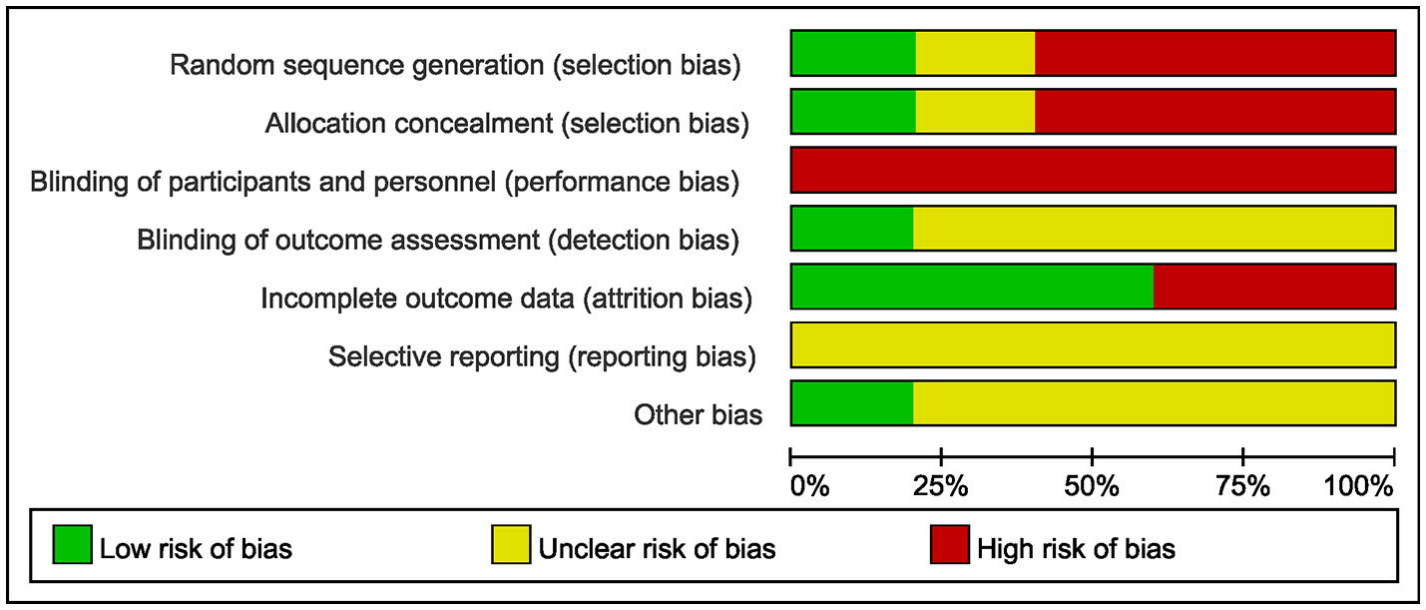
Risk of bias graph.

The methodological quality of the included studies was generally low. Because Tai Chi, as an intervention, cannot be blinded to the participants, the performance bias could not be ruled out. As three studies were N-RCTs, which did not carry out randomization, selection bias also existed. Furthermore, most studies did not provide enough descriptions to rule out the possibilities of detection and reporting bias (Table 2, Figure 2).

**Table 2:** Risk of bias assessment.

**Studies**	**Random sequence generation**	**Allocation concealment**	**Blinding of participants and personnel**	**Blinding of outcomes assessment**	**Incomplete outcome data**	**Selective reporting**	**Other bias**
Lan et al., 1999	High	High	High	Unclear	High	Unclear	Unclear
Chang et al., 2010	High	High	High	Unclear	Low	Unclear	Unclear
Wang et al., 2010	High	High	High	Unclear	High	Unclear	Unclear
Nery et al., 2012	Unclear	Unclear	High	Unclear	low	Unclear	Unclear
Nery et al., 2015	Low	Low	High	low	low	Unclear	Low

### Efficacy analyses

#### Tai chi vs. positive control

**Figure 3:**
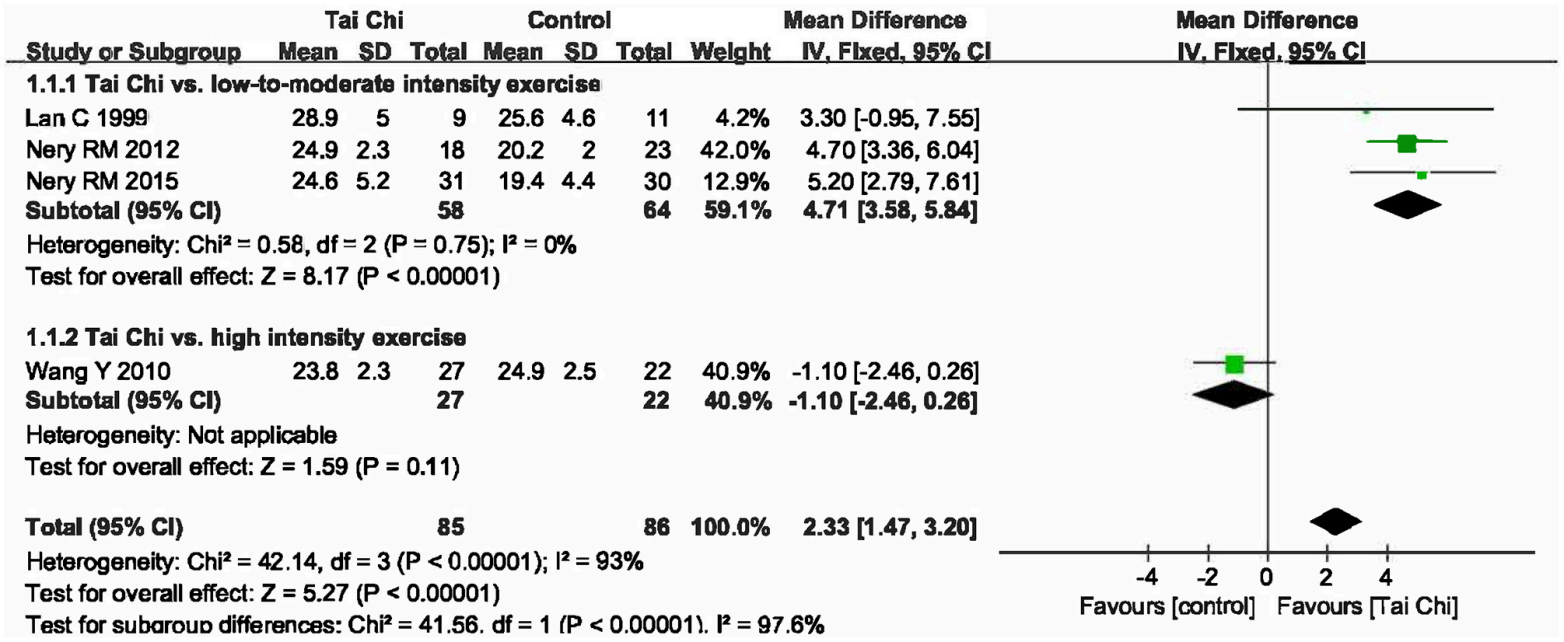
Forest plot of the comparison between Tai Chi and positive control for the outcome VO_2_max.

**Figure 4:**
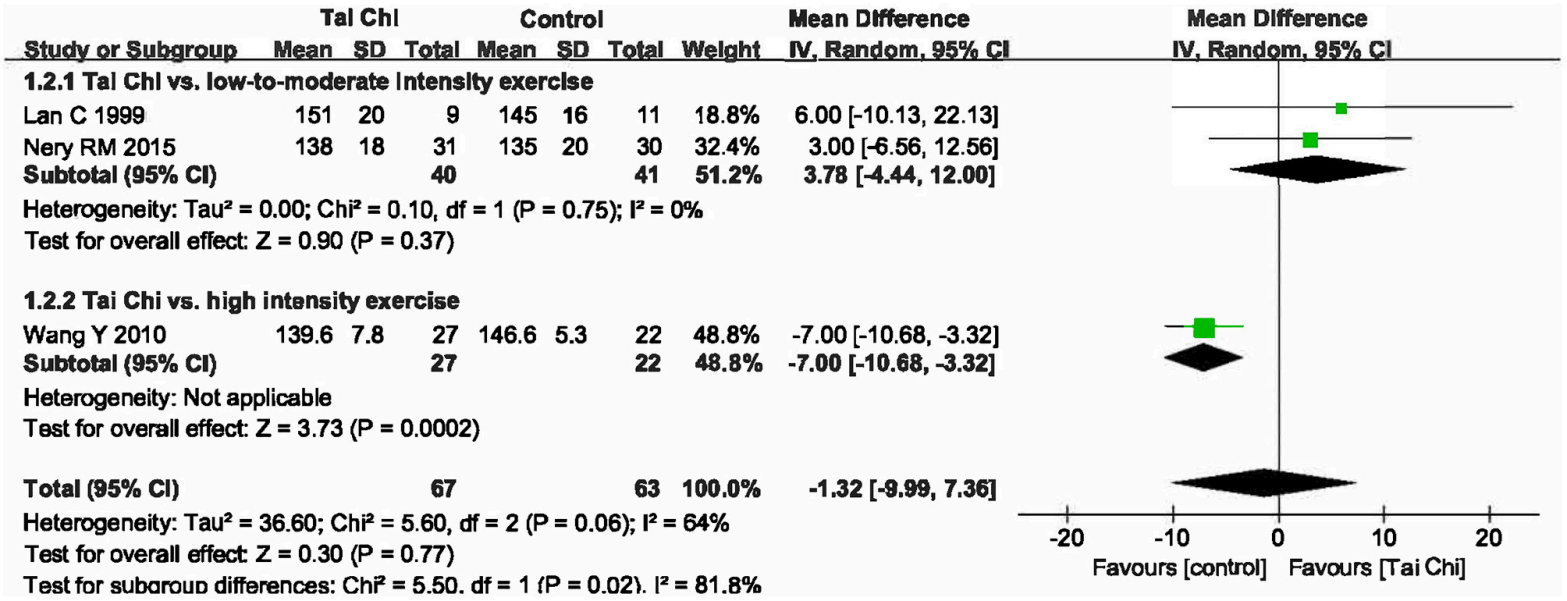
Forest plot of the comparison between Tai Chi and positive control for the outcome HR peak.

1. The effect on VO_2_max
  A total of four studies were included (Lan et al., 1999; Wang et al., 2010; Nery et al., 2012, 2015) with 171 patients, of whom 85 underwent Tai Chi intervention. Meta-analysis showed that there was significant heterogeneity among the studies (I^2^ = 93%, *P* < 0.00001). Therefore, we further conducted subgroup analyses according to different exercise intensity in the control groups. The in-group heterogeneity was small (I^2^ = 0%, *P* = 0.75), so we chose a fixed-effect model to do quantitative synthesis. Compared to other types of low-to-moderate intensity exercise, Tai Chi significantly improved VO_2_max in coronary disease [MD = 4.71, 95% CI [3.58, 5.84], *P* < 0.00001], while it tended to be less effective in improving VO_2_max compared to high-intensity exercise; this difference, however, was not statistically significant [MD = −1.10, 95% CI (−2.46, 0.26), *P* = 0.11] (Figure 3).
2. The effect on HR peak
  Three studies were included (Lan et al., 1999; Wang et al., 2010; Nery et al., 2015) with 130 patients, of whom 67 went through Tai Chi intervention. There was substantial heterogeneity among the included studies (I^2^ = 64%, *P* = 0.06), so we first performed quantitative synthesis using a random-effects model, which showed no difference between Tai Chi and other exercises on improving HR peak [MD = −1.32, 95% CI (−9.99, 7.36), *P* = 0.77]. Then, we performed subgroup analyses according to different exercise intensity in control groups. Compared to other types of low-to-moderate intensity exercise, Tai Chi showed no significant differences in improving HR peak [MD = 3.78, 95% CI (−4.44, 12.00), *P* = 0.37]; however, it did show reduced efficacy when compared to high-intensity exercise [MD = −7.00, 95% CI (−10.68, −3.32), *P* = 0.0002] (Figure 4).

#### Tai chi vs. blank

**Figure 5:**
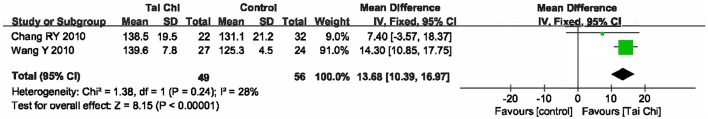
Forest plot of the comparison between Tai Chi and blank for the outcome HR peak.

1. The effect on VO_2_max
  There was only one trial comparing the effects of Tai Chi on VO_2_max in patients with coronary disease with those without exercise. The results showed that even in low intensity exercise, Tai Chi could significantly improve VO_2_max, and when exercise intensity was increased to moderate, VO_2_max improved further (Wang et al., 2010).
2. The effect on HR peak
  Two studies were included (Chang et al., 2010; Wang et al., 2010) with 105 patients, of whom 49 underwent Tai Chi intervention. The heterogeneity among the included studies was relatively small (I^2^ = 28%, *P* = 0.24); hence, we carried out quantitative analysis using a fixed-effect model that showed Tai Chi could greatly improve HR peak compared to no exercise [MD = 13.68, 95% CI (10.39, 16.97), *P* < 0.00001] (Figure 5).

### Adverse event

Of the five included studies, the initial recruitment had 305 patients with coronary disease. There were only 14 cases lost to follow-up, none of which were due to a major cardiac event, and we collected complete data of 291 cases (95.41%). Only one study reported adverse reactions, wherein there were 13 cases of adverse reactions in the Tai Chi group: two cases of dyspnea, five of muscle pain, and six of fatigue.

### Evidence evaluation

We used the GRADEpro online summary of findings table for outcomes to grade the available evidence. Tai Chi as an intervention cannot be blinded to the participants, and the population size of each meta-analysis was <400; hence, the evidence of each finding was graded as low level (Table 3).

**Table 3:** GRADEpro evidence grading.

**Quality assessment**						**Summary of findings**				**Quality**	**Importance**
						**No. of patients**		**Effect**			
**No. of studies**	**Limitations**	**Inconsistency**	**Indirectness**	**Imprecision**	**Other considerations**	**Tai Chi**	**Positive control**	**Relative (95% CI)**	**Absolute**		
**VO_2_ MAX–TAI CHI vs. LOW-TO-MODERATE INTENSITY EXERCISE (BETTER INDICATED BY LOWER VALUES)**											
3	Serious^a^	No serious inconsistency	No serious indirectness	Serious^b^	None	58	64	–	MD 4.71 higher (3.58 to 5.84 higher)	⊕⊕⊝⊝ LOW	CRITICAL
**VO_2_ MAX–TAI CHI vs. HIGH INTENSITY EXERCISE (BETTER INDICATED BY LOWER VALUES)**											
1	Serious^a^	No serious inconsistency	No serious indirectness	Serious^b^	None	27	22	–	MD 1.1 lower (2.46 lower to 0.26 higher)	⊕⊕⊝⊝ LOW	CRITICAL
**HR PEAK–TAI CHI vs. LOW-TO-MODERATE INTENSITY EXERCISE (BETTER INDICATED BY LOWER VALUES)**											
2	Serious^a^	No serious inconsistency	No serious indirectness	Serious^b^	None	40	41	–	MD 3.78 higher (4.44 lower to 12 higher)	⊕⊕⊝⊝ LOW	IMPORTANT
**HR PEAK–TAI CHI vs. HIGH INTENSITY EXERCISE (BETTER INDICATED BY LOWER VALUES)**											
1	Serious^a^	No serious inconsistency	No serious indirectness	Serious^b^	None	27	22	–	MD 7 lower (10.68 to 3.32 lower)	⊕⊕⊝⊝ LOW	IMPORTANT
**HR PEAK–TAI CHI vs. BLANK (BETTER INDICATED BY LOWER VALUES)**											
2	Serious^a^	No serious inconsistency	No serious indirectness	Serious^b^	None	49	56	–	MD 13.68 higher (10.39 to 16.97 higher)	⊕⊕⊝⊝ LOW	IMPORTANT

a *Tai Chi cannot be blinded as an intervention*.

b *Total population size is <400*.

## Discussion

Tai Chi is a traditional exercise in China and has been widely practiced since ancient times. In recent years, it has gained increased popularity in health care, especially among senior people (Hong et al., 2000; Li et al., 2008). Tai Chi has proven to be a low-to-moderate–intensity aerobic exercise. As cardiorespiratory fitness, or aerobic capacity, is one of the main concerns in cardiac rehabilitation, Tai Chi may have potential benefits in cardiac rehabilitation through the improvement of cardiorespiratory fitness.

### Summary of main results

The participants in our systematic review were all elderly patients with coronary disease, and our quantitative analysis showed that compared to no exercise or other types of low- or moderate-intensity exercise, Tai Chi can significantly improve VO_2_max. At the same time, the completion rate of all studies was above 95% with no severe adverse events, which suggests that Tai Chi might be an effective and safe choice for coronary disease rehabilitation by improving cardiorespiratory fitness (Yang et al., 2015). Tai Chi was less effective when compared to high intensity aerobic exercises, which may be explained by the fact that Tai Chi comprises slow, gentle, and graceful movements, as well as deep breathing and relaxation. Further, it is a complex, multi-component intervention that integrates physical, psycho-social, emotional, spiritual, and behavioral elements, and not just the utilization of oxygen (Sannes et al., 2008; Taylor-Piliae, 2014). Studies have proved that apart from improving vascular endothelial function and balancing cardiovascular risk factors such as blood lipid and glucose levels and blood pressure, Tai Chi can also regulate patients' mental status and improve sleep quality (Hambrecht et al., 2000; Yeh et al., 2008; Gellis and Kang-Yi, 2012; Hui et al., 2016; Wang et al., 2016; Alenazi et al., 2017). Besides, our review showed inconsistencies of Tai Chi's effect on HR between different control groups; hence, further studies are needed.

### Strengths and limitations

A previous meta-analysis showed that Tai Chi is effective in improving aerobic capacity in different groups of people (Taylor-Piliae, 2008). Two other previous meta-analyses about Tai Chi for coronary disease only included one study concerning VO_2_max; thus, both only performed a qualitative analysis to describe the effect of Tai Chi on VO_2_max (Ng et al., 2012; Nery et al., 2014). By using quantitative synthesis, our review firstly showed that Tai Chi can improve VO_2_max in patients with coronary disease, which further suggests that Tai Chi could be applied in cardiac rehabilitation as an adjuvant therapy to improve cardiorespiratory fitness. Moreover, we have published the protocol for this study in PROSPERO.

This review also has limitations. We only performed a search for Chinese and English studies, and it is possible that articles on Tai Chi for coronary disease rehabilitation may have been published in other languages. Moreover, in our review, the dose of Tai Chi practice and duration of cardiac rehabilitation were not considered, and different doses of exercise may have different effects. The methodological quality of the included trials was not promising: in addition to the fact that Tai Chi cannot be blinded, the included studies have other flaws such as poor randomization and lack of trial protocols. Because we only included five studies, we did not generate a funnel plot to detect any probable publication bias. Further, the GRADEpro showed a low level of available evidence.

### Implications for research

Although this study shows that Tai Chi may be effective and safe for coronary disease rehabilitation, the current evidence, and potential findings should be interpreted carefully because of poor methodological quality of these studies, insufficient evidence for efficacy and safety, and clinical heterogeneity.

Future studies should pay more attention to the effect of Tai Chi on VO_2_max in patients with coronary disease. Further rigorous RCTs with larger sample size and high methodology quality are required to explore the effects of Tai Chi intervention in clinical practice and provide evidence-based data for the promotion of Tai Chi.

## Conclusion

Compared to no exercise or other types of low-to-moderate intensity exercise, Tai Chi seems a good choice for coronary disease rehabilitation in improving cardiorespiratory fitness. However, due to the poor methodology quality, more clinical trials with larger sample size, strict randomization, and clear description about detection and reporting processes are needed to further strengthen this evidence.

## Author contributions

Conceived and designed the experiments: YY, YW, and SW. Performed the experiments: YY, YW, SW, PS, and CW. Literature searching: YY, PS, and CW. Data extraction and risk of bias assessments: YY, PS, and CW. Analyzed the data: YY, YW, and SW. Wrote the paper: YY. Read and approved the manuscript: YY, YW, SW, PS, and CW.

### Conflict of interest statement

The authors declare that the research was conducted in the absence of any commercial or financial relationships that could be construed as a potential conflict of interest.
